# Applying Principles of Regenerative Medicine to Vascular Stent Development

**DOI:** 10.3389/fbioe.2022.826807

**Published:** 2022-03-07

**Authors:** Prakash Parthiban Selvakumar, Michael Scott Rafuse, Richard Johnson, Wei Tan

**Affiliations:** University of Colorado Boulder, Boulder, CO, United States

**Keywords:** stent, selective regeneration, reendothelialization, restenosis, bioabsorable

## Abstract

Stents are a widely-used device to treat a variety of cardiovascular diseases. The purpose of this review is to explore the application of regenerative medicine principles into current and future stent designs. This review will cover regeneration-relevant approaches emerging in the current research landscape of stent technology. Regenerative stent technologies include surface engineering of stents with cell secretomes, cell-capture coatings, mimics of endothelial products, surface topography, endothelial growth factors or cell-adhesive peptides, as well as design of bioresorable materials for temporary stent support. These technologies are comparatively analyzed in terms of their regenerative effects, therapeutic effects and challenges faced; their benefits and risks are weighed up for suggestions about future stent developments. This review highlights two unique regenerative features of stent technologies: selective regeneration, which is to selectively grow endothelial cells on a stent but inhibit the proliferation and migration of smooth muscle cells, and stent-assisted regeneration of ischemic tissue injury.

## Introduction

Cardiovascular diseases, a group of disorders of the heart or blood vessels, are considered to be the leading cause of mortality worldwide. An estimated 17.9 million people died from cardiovascular diseases in 2019 (32% of all global deaths), with 85% due to heart attack and stroke (WHO, 2021). Coronary artery disease is the most common type of cardiovascular diseases; (Virani et al., 2020) about 18.2 million (6.7%) adults age 20 and older in US have this disease.

Angioplasty, a surgical procedure to open vessel blockages along with placement of a vascular stent, is a common treatment for heart attack and an option for stroke treatment or stroke prevention. More than 2 million people get a stent each year for coronary artery disease alone (Medtronic, 2019). Coronary artery stents account for just over two-thirds of all types of vascular stents. The global market of coronary stents was valued at an estimated $7.7 billion in 2019 and is expected to grow at a compound annual growth rate of 4.7% to reach $11.3 billion in 2027 (GVS, 2020), with some predicting even faster growth (9.8%) (DAIC, 2020). This growth is driven by the rising prevalence of cardiovascular diseases, a growing geriatric population, an increased number of angioplasties and rising preference for the procedure. Though stenting is suggested for severe narrowing of coronary arteries according to current clinical guidelines, a recent report questioned the effectiveness of stenting, compared to medications, in many patients with severe but stable heart disease (Reuters, 2019; NIH, 2020). Additionally, restenosis and/or other disease progression, after 4 years of stenting, were found in 26–35% patients in a study (Taniwaki et al., 2014; Cassese et al., 2015). In patients with peripheral artery stents, 18–40% at 12 months were reported to have in-stent restenosis (Schillinger et al., 2006; Laird et al., 2010), while 5–10% for patients with coronary stents (Shlofmitz et al., 2019; Lawton et al., 2021). To strategize for improved stent performances, there is a re-surging interest in resorbable biomaterials, surface and biomolecule engineering approaches, capitalizing on the concept of vascular healing and regeneration.

Now is an important moment to examine regenerative approaches for this widely-used device in interventional cardiology and assess their benefits and risks for future stent therapies. We will start with a brief overview of the current status of stent uses in clinic, including stent types and uses in treating various cardiovascular diseases. Then, this review focuses on new methods emerging in recent stent research endeavors related to the regeneration concept. Comparative analyses of these methods include the regenerative effects, therapeutic effects and challenges faced. Lastly, thoughts about future perspectives in this subject area are provided in context of recent developments in relevant regenerative medicine.

## Current Status of Clinical Use

Vascular stents are a device to treat a partially clogged artery by holding the arterial wall open and preventing it from collapsing. The majority of stents within the coronary stent market are bare metal stent (BMS) and drug eluting stent (DES), with the latter holding a larger share and being accepted as a safer treatment (Jensen and Christiansen, 2019). Nevertheless, the BMS is still widely used due to low cost and low hospitalization rates.

**FIGURE 1:**
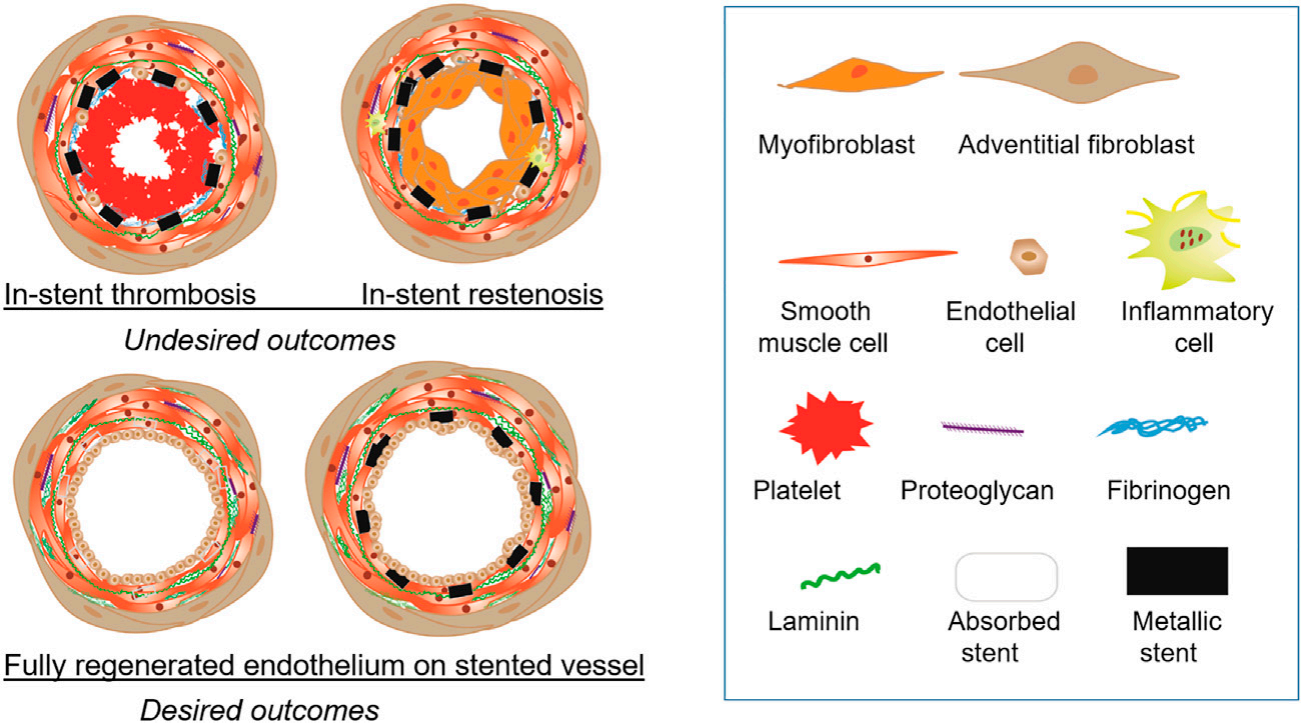
Outcomes of endovascular stents. (Top panel) Stents produce undesired outcomes such as in-stent thrombosis and in-stent restenosis. In-stent thrombosis involves accumulation of platelets and presence of fibrinogen. In-stent restenosis causes narrowing of the lumen greater than 50% due to the overgrowth of smooth muscle cells with neointima formation. (Botton panel) The ideal vascular regeneration with stents will have a fully developed endothelial monolayer lining the lumen with either the stent completely bioabsorbed or permanently encapsulated in the intimal layer.

Major clinical issues for stent usage are thrombosis and restenosis. Restenosis, re-narrowing of the stented vessel due to proliferation and migration of smooth muscle cells, is triggered by arterial injury during stenting. Figure 1 illustrates desired and undesired fates of a stented vessel. BMS shows a higher rate of restenosis than DES, while the risk of DES is often a very late (>12-month post-implantation) thrombosis. In general, the pathology associated with stent thrombosis and restenosis starts with the loss of endothelium due to injury, followed by fibrinogen absorption, platelet activation or neointimal hyperplasia (Nakamura et al., 2016). The contributing factors are numerous, including biological, patient-specific, mechanical, and technical factors. The evolution of stenting technologies is accompanied by the gradually improved understanding of stent-related vascular pathologies such as acute arterial injury, inflammation, extracellular matrix deposition, and negative vascular remodeling. It is only through addressing these relevant pathologies that new stent technology can be developed to balance acute, chronic, and long-term requirements for stability, healing and regeneration of stented vessels. The most important element is a complete regeneration of fully functional endothelium over stented vessels.

### DES

Widely-used DESs have a polymer coating blended with a drug or drug mixture that inhibit vascular smooth muscle cell growth. These anti-proliferative drugs are released over several months to help prevent restenosis. After that period, the stent may trigger platelet adhesion initiating thrombotic response. Controlled release of anti-proliferative agents inhibits neointimal growth but also delays arterial healing, thereby predisposing to a late risk of thrombosis. This high rate of late thrombosis (1–12 months post-implantation), demonstrated by the first generation stents, stimulated DES research for new generation stents in the last decade.

Contemporary second or third generations of stents are superior to the first generation in a number of clinical measures, including late stent thrombosis and strut coverage (Otsuka et al., 2014; Wiemer et al., 2017; Migita et al., 2020), showing a significant reduction in the risk of target lesion revascularization compared to early-generation DESs. Compared to the first generation stents, these stents display thinner stent struts (7–20 μm vs. 75–140 μm) and advancements in coatings including new anti-proliferative drugs and/or new polymers (Bangalore et al., 2018; Zaman et al., 2019). Reduced strut thickness offers greater flexibility and deliverability, while improvements in the drug or polymer realm permit faster vascular healing. Polymer-free stents are also available to decrease inflammatory reactions (McGinty et al., 2015; Worthley et al., 2017). Though newer generation DESs improve the effectiveness and safety profiles of their predecessors, long-term chronic inflammation and very late thrombosis remain serious concerns (Mori et al., 2017). More importantly, the prevention of cell growth by anti-proliferative drugs is counterproductive to vascular healing and regeneration; the coverage of stents with endothelial cells requires growth of these cells.

### Covered Stent

Covered stents have a fabric material component that covers a metal stent. The majority of covered stents use polytetrafluoroethylene and have various clinical applications in peripheral arterial disease management. Covered stents offer an effective strategy for the creation of transjugular intrahepatic portosystemic shunts (Geeroms et al., 2017; Cho et al., 2020), as well as for repair endovascular aneurysm (O’Donnell et al., 2019), traumatic arterial lesions, and obstructive vascular disease of the aortoiliac and femoropopliteal sectors. Recently, the application of covered stents have expanded to seal coronary artery perforation (Lee et al., 2016; Wang et al., 2017; Birkemeyer et al., 2021; Wańha et al., 2021) and other perforations in clinic (Rizk et al., 2020). It also has a potential role in the treatment of friable embolization-prone plaques (Dogu Kilic et al., 2016). In the case of transjugular intrahepatic portosystemic shunts, covered stents, compared to BMS, showed higher estimated overall survival in patients (Gupta et al., 2017).

Recently, covered stents have become a platform that offer a minimally invasive, safe, and effective solution for arterial emergencies, such as bleeding, pseudoaneurysm, dissection, or fistula (Geeroms et al., 2017; O’Donnell et al., 2019). During the last few decades, treatment of these potentially life-threatening lesions has shifted from emergency open surgery to a stenting approach. Focusing on iatrogenic arterial injuries, Ruffino et al. gave an insight into the advantages of covered stents (Ruffino et al., 2020). In fact, it was believed that almost all arterial lesions may be treated with covered stents, except for those without anatomic suitability.

Taken together, current stent-assisted therapies—BMS, DES and covered stents—are employed to combat a wide range of vascular diseases and emergencies. Despite low complication rates (approximately 2–3% per year after the first year), stent therapy is still associated with a risk of restenosis and thrombosis, which persists for over 20 years. Recent comparisons of stent therapy and non-stent therapy for treating different clinical conditions such as coronary artery disease and infrainguinal peripheral artery disease showed no difference between stent and non-stent therapies, while the stent procedure is more invasive and has a significantly higher cost (Banerjee et al., 2019; NIH, 2020). Therefore, to demonstrate more competitive advantages over non-stent artery interventions, stent-assisted therapies demand innovations to proactively enhance arterial regeneration and long-term arterial health, in addition to the prevention of disastrous outcomes.

## Novel Developments and Challenges in Stents

Similar to the application of regenerative medicine concepts in other tissues or devices, regeneration-relevant approaches emerge in the stent technology in two forms: regenerative surface and biodegradable or bioresorable scaffolding materials. However, due to the presence of diseased artery tissue around a stent, major considerations of all stent technologies must include their therapeutic impact such as the inhibition of arterial recoil and smooth muscle proliferation. To this end, we review the two major types of approaches to stent design by comparing and evaluating their regenerative effects and therapeutic effects.

### Regenerative Stent Surface

Continued efforts towards stent surface modifications are seen in the 1990s and 2000s as well as more recently. Earlier efforts focused on therapeutic effects such as reduced restenosis, using 1) *inorganic coatings*, including diamond-like carbon (Airoldi et al., 2004), pyrolytic carbon (Danzi et al., 2004), titanium nitride oxide (Windecker et al., 2005), and carbide (Unverdorben et al., 2003); 2) *gene-eluting coatings*, incorporating mRNA, siRNA, miRNA, or plasmid DNA of therapeutic genes in the coating; (Feldman et al., 2000; Sharif et al., 2012; Wang et al., 2021) 3) *cell-seeded stent,* using endothelial cells or progenitor cells (Zhu et al., 2008; Shi et al., 2010; Jantzen et al., 2011; Raina et al., 2014). The inorganic coatings provided ineffective or inconclusive performance (e.g., restenosis rate), compared to uncoated BMS (Kim et al., 2005; Meireles et al., 2007; O’Brien and Carroll, 2009). The gene-eluting coatings, like DES, incorporate genes for therapeutic moiety, but offer a longer term efficacy and a wider variety of therapeutic strategies beyond suppressing cell proliferation for restenosis inhibition (Yang et al., 2013; Adeel and Sharif, 2016; Fishbein et al., 2017; Hytönen et al., 2018). Nevertheless, the clinical translation of such coatings is still extremely challenging due to significant technical hurdles, demanding improvements over release kinetics, cell-specific transfection, reduced toxicity, and inflammation. Similarly, great difficulty in the clinical translation is faced by cell-seeded stents (Jantzen et al., 2016; Tsukada et al., 2021).

The root cause of stent pathology, as revealed by numerous studies on BMS and DES, (Cornelissen and Vogt, 2019; Bedair et al., 2017; Kobo et al., 2020; Scafa Udriște et al., 2021), is endothelial dysfunction and/or impaired growth of endothelial cells in the stented artery. Healthy endothelial cells naturally prevent the blood from clotting, and produce molecules such as nitric oxide (NO) and prostacyclin for smooth muscle relaxation and growth inhibition. Thus, modifications of the stent surface in the last decade involve approaches to promote *in situ* re-endothelialization or to integrate critical endothelial functions. This is achieved through cell secretomes, cell-capture coatings, mimics of endothelial products (e.g., NO), surface topography, endothelial growth factor, cell-adhesive peptides and other biologics. Table 1 provides a brief summary of these functionalization approaches. Details of each approach are described in following paragraphs. As reviewed by Bedair et al. (2017), the immobilization of molecules onto the stent surface involves physical adsorption, encapsulation, ionic bonding or covalent conjugation through various chemical reactions such as carbodiimide chemistry. The covalent conjugation may begin with plasma treatment or a layer of polydopamine for abundant free hydroxyl groups, and then attach a functional polymer such as bioclickable polymer.

**TABLE 1:** A summary of regenerative surface modification approach from the literature.

Regenerative approaches	Examples	Animal model for evaluation	Time points	Regenerative outcome	Therapeutic outcome	Challenges/Questions
Stem cell secretomes	Exosome-eluting stent using MSC-derived exosomes	Bilateral renal ischaemia-reperfusion injury model with rat (Hu et al., 2021)	Short term (28 days)	Accelerated re-endothelialization; promoted muscle tissue repair through increased reperfusion	Decreased in-stent restenosis; regulated macrophage polarization	Isolation of exosomes that are free from harmful contaminants and have a consistent set of functional properties
EPC-capture molecules	Anti-CD133 and Anti-CD34	Rabbit abdominal aorta (Wu et al., 2015)	Medium term (12 weeks)	Better EPC capture by anti-CD133 stent compared to anti-CD34 stent	Anti-CD133 stents accelerate tissue regeneration without excessive neointima	A low number and high heterogeneity of circulating EPC
	Anti-VE-Cadherin and Anti-CD34	Rabbit iliac artery (Lee et al., 2012)	Short term (42 days)	Better EPC capture and re-endothelialization on anti-VE-Cadherin stent compared to anti-CD34 stent	Anti-VE-Cadherin stents reduced restenosis *in vivo*	
	Anti-CD34	Pig coronary artery (van Beusekom et al., 2012)	Medium term (90 days)	Improved early endothelialization	No significant difference in neointimal thickness with anti-CD34 stent	Cannot differentiate between EC produced from circulating cells and remnant EC proliferation
	Anti-CD133	Pig left anterior or circumflex arteries (Sedaghat et al., 2013)	Short term (28 days)	No effective increase in re-endothelialization or neointima reduction	No significant therapeutic outcome	No regenerative or therapeutic benefits. Larger sample size, longer term studies are needed
	Combo^®^ DTS (anti-CD34)	Human patients (Blessing et al., 2020)	Long term (615 days) with median follow-up of 189 days	Augmented EPC recruitment due to anti-CD34 antibody labeling might promote neointima formation.	Combo^®^ DTS showed 40% restenosis rate with low rate of major cardiovascular events in follow-up	Adverse EPC differentiation in the proinflammatory environment and/or enhanced attraction of myeloid cells may cause restenosis.
	Anti-CD133 with chitosan/hyaluronic acid coated stent	Pig coronary artery (Zhang et al., 2015)	Short term (28 days)	Better resistance to blood flow erosion; Targeted capture of hematopoietic stem cells; Inhibited migration and proliferation of smooth muscle cells	Improved re-endothelialization and reduced thrombosis, inflammation and rejection	The role of chitosan and hyaluronic acid on the improved stent performance is unknown to be studied
	VE-cadherin extracellular domain + adhesive protein	Rat (Yang et al., 2020a)	Short term (1 month)	Accelerated endotheliali-zation; Tight junction formation; Improved endothelial barrier function	Good hemocompatibility	Will the regenerated endothelial layer with tight junctions prevent late stage thrombosis?
NO-producing coatings	NO donor: NONOate	Rabbit (Zhu et al., 2020)	Short term (1 month)	Accelerated regeneration of endothelial cells	Anti-restenosis; Good anticoagulation	NO availability is for a limited time; long-term studies are needed
	NO donor: DETA NONOate	Pig coronary artery (Elnaggar et al., 2016)	Short term (28 days)	NO release for 5 days; Induce endothelialization	Reduced inflammation score; Lowered fibrinogen adsorption; Inhibited neointimal hyperplasia	Longer NO release is needed; Identifying appropriate NO release molecule is challenging
	NO-generating SeCA-Dopamine	Rabbit iliac artery (Yang et al., 2018)	Medium term (3 months)	Promoted reendotheliali-zation; NO release supported competitive growth of HUVECs over HUASMCs	Reduced in-stent restenosis and neointimal hyperplasia	The integrity of the endothelial monolayer with sustained NO release must be investigated. The long-term biocompatibility of the Cu- or Se- catalyst is important to the translation of this approach into the clinical practice
	NO-generating: Nano Cu	Rabbit iliac artery (Fan et al., 2019)	Short term (4 weeks)	Promoted re-endothelialization	Promoted anticoagulation and anti-hyperplasia; suppressed thrombosis and stent restenosis	
	NO generating Organoselenium (SeCA)	Rabbit iliac artery (Yang et al., 2020b)	Medium term (12 weeks)	Rapid re-endothelialization; SMC migration and proliferation, EPC recruitment	Inhibition of thrombosis, and effective in-stent restenosis prevention	
Soluble growth factors	rhVEGF	Rabbit iliac artery (Van Belle et al., 1997)	Short term (28 days)	Accelerated endothelialization within 7 days	Reduced in-stent intimal thickness	Formation of mural thrombus should be prevented
	VEGF	Pig coronary artery (Takabatake et al., 2014)	Short term (14 days)	Provided highly selective capture of EPCs, when compared with anti-CD34 antibody-bound stents; Rapid formation of intact endothelium		Results may depend on the form and binding of VEGF. Long-term studies are needed
	Rapamycin-VEGF coating	Pig artery (Wang et al., 2020b)	Short term (42 days)	This combination promoted growth of EC over SMC; efficient re-endothelialization	Suppression of in-stent restenosis	
Surface pattern	Nanotexturing	Rabbit iliac artery (Cherian et al., 2020)	Short term (8 weeks)	Preferential proliferation of endothelial cells over smooth muscle cells; complete endothelial coverage	Reduced neointimal thickening and in-stent restenosis	Unknown mechanism underlying selective cell proliferation on nanostructures
Adhesive Peptides	cRGD	Pig coronary artery (Blindt et al., 2006)	medium term (12 weeks)	Early recruitment of EPC by αvβ3-integrins; Accelerated endothelialization	Reduced neointimal area and percent area stenosis	Attracting the right kind of EPC from the blood stream is a challenge
	RGD and CXCL1	Mice carotid artery (Simsekyilmaz et al., 2016)	Short term (1 week)	Adhesion of early angiogenic outgrowth cells, a type of EPC; Increased re-endothelialization	Reduced neointima and thrombus	Extend this work to long term to firmly establish the efficacy of this process
	REDV	Rabbit iliac artery (Wei et al., 2013)	Short term (28 days)	Random and tightly arranged endothelial cells	Significantly inhibited neointimal hyperplasia	Is REDV a stand-alone peptide in re- endothelialization?
	WKYMVm-HA + sirolimus	Rabbit iliac artery (Jang et al., 2015)	Short term (6 weeks)	Consecutive endothelial lining	Low restenosis rate, similar to commercial DES	Lack of specificity of cell attachment
Other approaches	Epigallocatechin gallate/copper	Rabbit abdominal aorta (Zhang et al., 2021)	Short (1 month) and medium term (3 months)	Upregulated VEGF; *In-situ* re-endothelialization	Anti-hyperplasia; Suppressed SMC proliferation and migration; Enhanced anticoagulation; Alleviated inflammatory reactions	The quality and integrity of the endothelial monolayer needs to improved, which is a major challenge
	Heparin/SeCA	Rabbit iliac artery (Qiu et al., 2019)	Short (1 month) and medium term (3 months)	Created environments that favored the growth of endothelial cells compared to smooth muscle cells	Anti-thrombogenic, anti-restenosis	The intimal hyperplasia and in-stent restenosis parameters soared from 1 month to 3 months. What will be the long term effect on these parameters?
	Biodegradable stent (Polylactic acid)	Pig coronary artery (Lee et al., 2019)	Short term (28 days)	Widest lumen area; Rapid EC proliferation	Less neointimal hyperplasia with no atherosclerosis or thrombosis	The rate of biodegradation matches the rate of formation of neoartery
	CD31-mimic	Pig coronary artery (Diaz-Rodriguez et al., 2021)	Short term (28 days)	Full endothelialization with no activated platelets/leukocytes	Normal arterial media with no thrombosis	Stability of the coating

HA, hyaluronic acid; SeCA, selenocystamine.

The regenerative aspects of a stent are indeed unique. Two prominent features are noted. First, stent-related regeneration is “*selective regeneration*”, which is to selectively grow endothelial cells on the stent surface but inhibit the proliferation and migration of smooth muscle cells. Therefore, multifunctional coatings in recent developments exhibit both regenerative effect on the endothelium and therapeutic effects on the smooth muscle. Second, stent-assisted regeneration of ischemic tissue injury is needed in certain applications such as myocardial infarction and renal ischemia, where narrowing of an artery is accompanied by the loss of distal vasculature and thus tissue injury. Stenting provides an opportunity to regenerate the tissues with biologics that regenerate stented artery and distal vasculature (d’Souza et al., 2017). Regenerative biologics can also alleviate the impaired vasorelaxation in nonstented proximal and distal segments of stented arteries, in particular those with DES (Nakamura et al., 2011; van den Heuvel et al., 2010; Lim et al., 2012).

#### Stent Incorporated With Cell Secretome

Cardiovascular stents incorporated with secretomes from stem cells represents a novel direction for regenerative stents. Hu et al. recently reported exosome-eluting stents for vascular healing and tissue regeneration after ischemic injury (Hu et al., 2021). These stents were coated with exosomes derived from mesenchymal stem cells. The bioactive stents promoted vascular healing and repair of ischemic condition, which usually need additional procedures for regenerative therapy in a patient. Exosomes are nanoscale membranous sacs secreted by most cell types; those from stem cells show great potentials of delivering regenerative and therapeutic benefits (Burke et al., 2016; Eisenstein, 2020; Nikfarjam et al., 2020; Wei et al., 2021). A major regenerative benefit of exosomes is to rejuvenate endothelial cells (Baruah and Wary, 2020), which can not only help to cover the stent with a healthy endothelium but also promote distal revascularization for tissue repair. A prominent therapeutic benefit of exosomes is immune-modulation or immune-suppression (Domenis et al., 2018; Suh et al., 2021). The exosome coating on stents may help camouflage the stent, blocking adverse immune reactions. Another novel aspect of the stent design by Hu et al. is the controlled release of exosomes from the stent coating in the presence of reactive oxygen species, a hallmark of inflammation (Hu et al., 2021). In the long run, stents incorporating cell products like exosomes or other forms of cell secretomes might offer a safer and simpler alternative to cell-seeded stents.

#### Cell-Capture Stent

Another alternative to cell-seeded stents is to utilize biomolecule coatings to capture circulating cells in the blood for endothelial regeneration (Sethi and Lee, 2012; Pang et al., 2015). To that end, various antibodies recognizing endothelial progenitor cell (EPC), like anti- CD34, VE-cad, or CD133, were loaded to the stent surface (Lim et al., 2011; Leopold, 2013; Wawrzyńska et al., 2019). These antibody-coated stents were compared in terms of their capability of capturing circulating EPCs. All types of antibody-coated stents were completely covered with a cell layer in earlier stage (e.g., one-week post-implantation) than BMS in animals. Animal studies showed that stents coated with anti-CD133 or VE-cad, compared with those with anti-CD34, accelerated re-endothelialization and reduced in-stent restenosis (Lee et al., 2012; Wu et al., 2015).

Preclinical or clinical results using cell-capture stents, however, are not always positive. Those with anti-CD34 were found to improve early endothelialization in swine but not affecting neointimal thickness (van Beusekom et al., 2012). Sedaghat et al., using a porcine model, found no difference in re-endothelialization or neointima formation with the use of CD133-stents compared with BMS (Sedaghat et al., 2013). This is supported by studies showing circulating EPCs or bone marrow-derived cells failed to significantly contribute directly to endothelial regeneration; cell-capture stent might increase restenosis rate in clinic (Blessing et al., 2020; Evans et al., 2021). Such inconsistency among preclinical outcomes of cell-capture stents can be attributed to the low number and the high heterogeneity of circulating EPCs (Medina et al., 2017; Keighron et al., 2018). Antibodies on stents may also increase unspecific binding of mononuclear cells, likely causing complications (Sedaghat et al., 2013).

For all regenerative purposes and translational intents, current and future endeavors utilizing cell-capture mechanism may continue in three directions: 1) combining cell-capture molecules with other prohealing/proregenerative molecules; (Chen et al., 2015; Zhang et al., 2015; Wang et al., 2020a; Yang et al., 2020a) 2) using more selective or specific molecules to recognize a subset of EPCs; (Hirase, 2016; Tang et al., 2016) 3) exploiting EPC-derived secretomes such as those in the form of exosomes (Zeng et al., 2021).

#### NO-Producing Coatings

NO is one of the most potent molecules produced by healthy endothelial cells that play multiple essential roles in cardiovascular physiology (Napoli et al., 2006; Strijdom et al., 2009). Vascular injury, just like in the case of stent deployment, reduced NO production of endothelium. Incorporating a NO-producing mechanism into stent coatings has a range of benefits, including the prevention of thrombosis through inhibiting platelet aggregation, anti-inflammation through reducing monocyte adhesion, inhibition of SMC migration and proliferation through SMC relaxation, as well as stimulation of endothelial proliferation for stent endothelialization (Rao et al., 2020). A recent review by Rao et al. detailed this topic, in particular the therapeutic effect of NO-producing coatings for the prevention of thrombosis and restenosis (Rao et al., 2020).

There are two types of NO-producing coatings, NO-releasing and NO-generating coatings. NO-releasing coatings involves exogenous NO donors such as N-diazeniumdiolates (i.e., NONOates), S-nitroso-N-acetylpenicillamine, and peptide amphiphiles (Kushwaha et al., 2010; Zhu et al., 2020), immobilized by polymer or liposome (Elnaggar et al., 2016). These donors release NO in limited periods of time. To extend the release time, recent developments involves the attachment of NO donors to polymers (Hopkins et al., 2018) or enzyme-sensitive linkers (Winther et al., 2018; Midgley et al., 2020). NO-generating coatings utilize eNOS gene (Sharif et al., 2012), or immobilize catalysts such as copper and selenium to convert endogenous NO donors such as S-nitrosothiols into NO (Yang et al., 2018; Fan et al., 2019; Zhang et al., 2019; Yang et al., 2020b). All the studies using stents with either NO-releasing or NO-generating coating show promising results *in vitro* and *in vivo*. This is consistent with the results from recent graft study which employed NO-producing vascular graft on rodents for 12 weeks and found rapid endothelialization and hampered SMC proliferation (Enayati et al., 2021). Despite promising outcomes, long-term preclinical evaluations on NO-generating stents are yet to be performed.

#### VEGF-Induced Regeneration

VEGF is a most potent, endothelial-specific growth factor to regenerate endothelial cells. Early attempts to incorporate it into stent coatings were made as early as 1990s (Van Belle et al., 1997). However, inconsistent results regarding re-endothelialization and inhibition of restenosis were found in animal studies, which might be derived from different methods of preparing VEGF-eluting stents. Opposite to the finding from an earlier study using VEGF-eluting stent (Van Belle et al., 1997), Swanson et al. found VEGF-eluting stents did not accelerate re-endothelialization or inhibit restenosis, but reduced stent thrombosis (Swanson et al., 2004). Both studies evaluated stents with iliac artery model on New Zealand rabbits. Recent VEGF-bound stents have found more success in the preclinical evaluations. Using a porcine coronary model, Takabatake et al. used VEGF on poly-(ethylene-co-vinyl alcohol) coated stents, and found VEGF-bound stents, compared with anti-CD34 antibody-bound stents, provided highly selective capture of EPCs, followed by a rapid formation of intact endothelium at an early period of stenting (Takabatake et al., 2014). Yang et al. showed DES with VEGF gene completed re-endothelialization and significantly suppressed in-stent restenosis after 1 month compared to commercial DES (Yang et al., 2013). Wang et al. spatially bound VEGF and rapamycin to the base and top of hierarchical capillary coating, respectively, and showed the competitive growth of endothelial cells over smooth muscle cells on the stent surface as well as a high level of re-endothelialization and a very low level of in-stent restenosis using a minipig model (Wang et al., 2020b).

In conclusion, VEGF-bound stents hold great promise in endothelial regeneration but the outcome using this regenerative molecule depends on: 1) co-grafting condition, such as drugs or other molecules coexisting on the stent and collaborating into action at the stent-artery interface; 2) the form of VEGF (e.g., gene, protein, and peptide), and 3) the conjugation method of VEGF, for example eluting vs. polymer-bound.

#### Surface Pattern

Specialized micro- and nano-patterns have been introduced on the stent surface to reduce the risk of blood clotting, expedite healing and improve endothelialization. The design of textures on the thin struts of a stent includes nanotubes, ridges, pores, diamonds, leafy structure, or even a pattern mimicking the shape of smooth muscle cells (Cherian et al., 2021) (Junkar et al., 2020). Importantly, the textures are assessed by preferential growth of endothelial cells over smooth muscle cells (Cherian et al., 2020). However, the reason for this selective cell proliferation remain elusive. Manufacturing methods can involve anodization (Saleh et al., 2017), atomic layer deposition (Yang et al., 2019), lithography or femtosecond laser. Lee and Desai, for example, altered the anodization conditions to achieve nanotubular coatings with 110 and 70 nm nanotube diameters, and found competitive growth of endothelial cells over smooth muscle cells on nanotubular stents (Lee and Desai, 2016). Nanostructure formation is free of polymers or new chemicals, which might speed up the approval process for the clinical translation.

#### Other Biologics-Based Coatings

Two major groups of other biologics used in stent coatings, often in combination with other approaches, are adhesive peptides and glycosaminoglycan (GAG) molecules such as heparin and hyaluronic acid or GAG-mimics such as chitosan.

Adhesive peptides are instrumental for the attachment of circulating cells like EPCs. Blindt et al. found that DES with cyclic RGD peptide inhibited neointimal hyperplasia by recruiting EPCs in porcine coronary arteries (Blindt et al., 2006). With improved vascular healing in second-generation DESs, recent approaches employed adhesive peptides in conjunction with other functional molecules such as CXCL1 (Simsekyilmaz et al., 2016), or more specific adhesive peptides such as YIGSR (Alexander et al., 2018) and REDV (Wei et al., 2013). Jang et al. coated DES with WKYMVm peptide and hyaluronic acid, and using rabbit iliac model found the WKYMVm coating promoted endothelial healing but did not reduce restenosis rate compared to commercial DES (Jang et al., 2015). Wei et al. immobilized REDV peptide and phosphorylcholine on BMS and showed the competitive ability of endothelial cell growth over smooth muscle growth *in vitro* and *in vivo*, and reduced restenosis compared to BMS (Wei et al., 2013). The downside of using adhesive peptides to recruit circulating cells, however, is its possibility of increasing unspecific binding of other circulating cells such as mononuclear cells.

Immobilization of GAGs or GAG-mimics onto the stent surface is a strategy to enhance stent anti-thrombogenicity. Heparin, heparin-like molecules and hyaluronic acid are often utilized in combination with other molecules to coat stent surfaces. Heparin was used with NO-producing, DES, or bioresorbable stents (Bae et al., 2017; Lee et al., 2019; Qiu et al., 2019; Zhu et al., 2020; Zhang et al., 2021). Due to its anti-thrombogenic, anti-fouling characteristics, hyaluronic acid often serves as a base adhesive material on stents (Zhang et al., 2015; Kim et al., 2016).

Besides adhesive peptides and GAGs, new signaling molecules such as CD31-mimic are used to stimulate the proliferation of adjacent endothelial cells to regenerate the endothelium (Diaz-Rodriguez et al., 2021).

#### Stent Coating With a Combination of Biomolecules

There is an increasing interest in researching multifunctional stent surfaces that contain components promoting therapeutic effect, selective regeneration, and/or critical endothelial functions. In particular, coatings are designed to both counteract endothelial dysfunction and mimic endothelial characteristics. For example, recent studies have combined NO production with cell-adhesive ligands or anti-thrombogenic molecules (Qiu et al., 2019; Yang et al., 2020b; Yang et al., 2020c; Lyu et al., 2020; Zhu et al., 2020; Ma et al., 2021; Zhang et al., 2021). Yang et al. functionalized the stent with NO-generating organoselenium and EPC-targeting peptide through mussel adhesive chemistry and bio-orthogonal conjugation (Yang et al., 2020b). The ratio of the two components on stent the coating was optimized for anti-thrombosis, smooth muscle inhibition and EPC-capture capacity, ultimately leading to rapid re-endothelialization and effective stent restenosis prevention *in vivo*.

#### Regenerative Strategies for Covered Stents

Few attempts have been made to functionalize or replace the existing polytetrafluoroethylene-covered stent. Among them is the use of polyurethane-covered stent in recent studies, but its clinical efficacy is yet to be demonstrated with comparisons to polytetrafluoroethylene-covered stent (Hernández-Enríquez et al., 2018; Song et al., 2021). A new trend is to develop covered stents using natural-derived or biodegradable materials with regenerative potentials, as illustrated by endothelial progenitor cells-laden coronary stent covered with ECM (Park et al., 2021) and stent covered with silk and elastin proteins (Putzu et al., 2019). Additionally, to replace inert materials used for covering stents, a wide range of vascular-regenerative polymers available for vascular graft applications can also be excellent candidates (Leal et al., 2021). For example, we demonstrated vascular grafts composed of coaxial fibers with a structure of polycaprolactone core and photoclickable, 4-arm thiolated polyethylene glycol-norbornene sheath, which can be conjugated to biomolecules for vascular regeneration (Iglesias-Echevarria et al., 2021). In the future, the platform of covered stent with regenerative covering may evolve into a new type of stent therapy which utilize bioabsorbable materials with regenerative signals to better treat vascular diseases.

**FIGURE 2:**
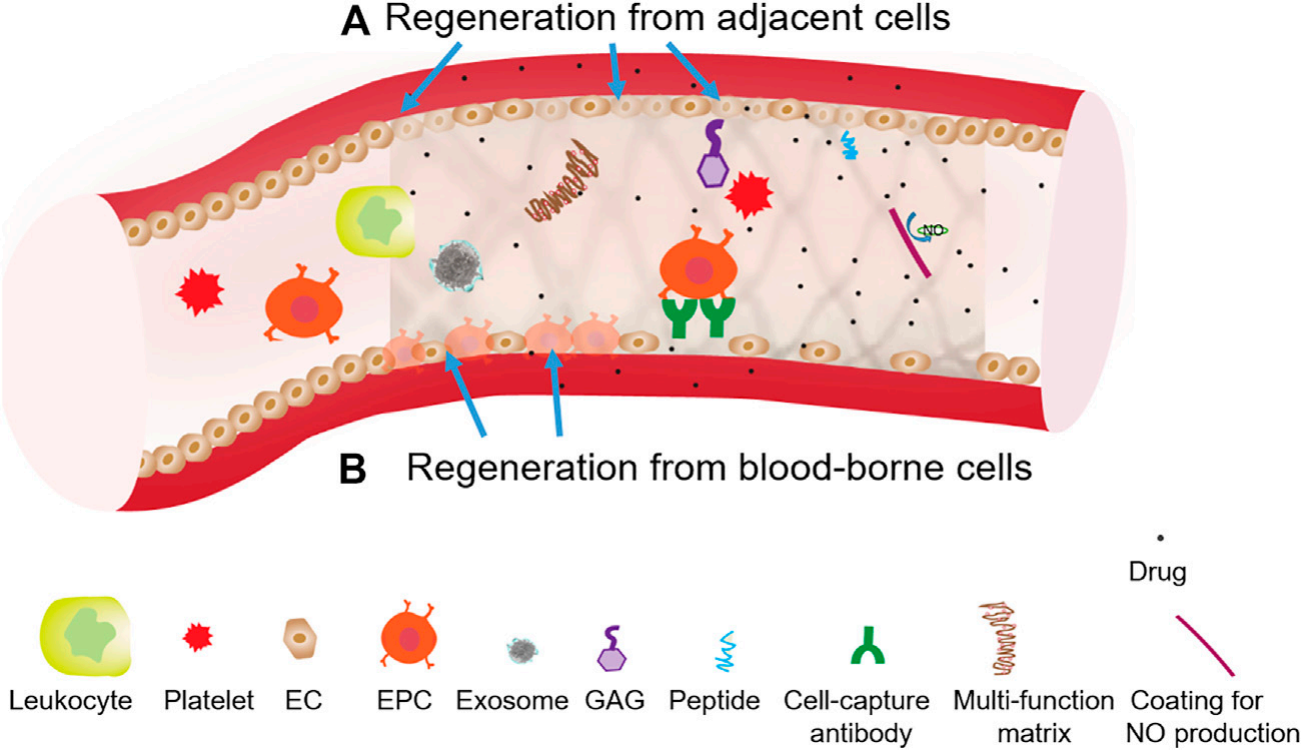
Two generation mechanisms using surface functionalizations to override drug inhition effects on stent endothelialization. **(A)** Regeneration from adjacent cells, which can involve the functionalization of stents with angiogenic peptide or NO producing coating. **(B)** Regeneration from blood-borne cells, which can involve the functionalization of stents with certain cell-capture antibodies, adhesive peptide or growth factor.

In summary, new developments in the stent surface functionalizations focus on the regeneration of the vascular endothelium or the restoration of major endothelial functions such as anti-thrombogenicity and NO production. The endothelium normally controls smooth muscle activities, and provides an efficient barrier against thrombosis and inflammation. However, the endothelium reestablished after the stenting procedure (e.g., percutaneous coronary intervention) is incompetent in terms of its integrity and function, showing poorly formed cell junctions, reduced expression of anti-thrombotic molecules (e.g., thrombomodulin, prostacyclin), and decreased NO production (Otsuka et al., 2012). Drugs from DES further inhibit endothelial regeneration or function restoration. The missing or incompetent endothelium in existing stent products (e.g., BMS, DES, and covered stent) is a root cause of late or very late in-stent thrombosis and restenosis, as well as impaired function of proximal or distal blood vessels. As shown in Figure 2, re-endothelialization mechanisms include regeneration from adjacent cells and regeneration from blood-borne cells, both of which require regenerative cues on stents to override the cell inhibition from therapeutic agents (i.e., drugs). Improved understanding of regeneration mechanisms for competently functioning endothelium is essential for long-term stent performances.

### Bioabsorbable Vascular Stent

To mitigate the risk of restenosis and thrombosis in stent, it has long been envisioned that a fully bioresorbable polymer is used in replacement of metal. The first-generation, FDA-approved BVS product was created and launched by Abbott as Absorb BVS stent in September 2012. This type of products was characterized by a degradation rate, ranging from 6 months to 2 years, to avoid undesired long-term effects, but demonstrated worse clinical performances including in-stent thrombosis, restenosis, and target vessel myocardial infarction, when compared to BMS or DES (Capodanno et al., 2015; Lipinski et al., 2016; Serruys et al., 2016). Thus, it was not recommended (Neumann et al., 2019), and pulled off the market in 2017. The first-generation BVS also was found a long-term concern on side branch ostia (Onuma et al., 2017).

Newer BVS technologies and devices mostly loaded with drugs are currently undergoing preclinical and clinical testing (Ellis et al., 2017; Serruys et al., 2017; Omar and Kumbhani, 2019). A promising feature of BVS, shown in a clinical study comparing BVS to DES, is more significant outward remodeling (Serruys et al., 2017). To decrease BVS thrombogenicity, one advancement, like the major improvement made in the second generation of DES, is a progressive reduction of the BVS strut thickness to as thin as 60 μm (Buccheri and Capodanno, 2019). The majority of BVSs on the pre-market consist of polymers such as polylactic acid used in Absorb BVS, but degradable metals such as magnesium (Chen et al., 2019; Hideo-Kajita et al., 2019) or zinc (Fu et al., 2020) are also actively being developed for newer generation of BVS.

To enhance therapeutic and regenerative performances of BVS, bioactive molecules can also coat the BVS surface or be integrated into the bulk polymer. Any regenerative molecules reviewed above (Table 1) may be added into or on top of BVS. Besides these molecules, Yang et al. formed a multilayer coating of collagen type III and hyaluronic acid on a polylactic acid stent via layer-by-layer assembly, which enhanced endothelialization and thromboprotection, and inhibited excessive neointimal hyperplasia using a rabbit abdominal aorta model (Yang et al., 2021). Collagen type III does not present binding sites for platelets while retaining the affinity for endothelial cells.

For a more detailed summary of BVS, please refer to additional reviews on the topic (Omar and Kumbhani, 2019; Cockerill et al., 2021; Yuan et al., 2022). In brief, researchers in academia and industry share the dream of a bioabsorbable scaffold that delivers therapeutic and regenerative agents to the vessel, maintains radial strength for a sufficient period of time (>6 months), and then disappears when therapy and regeneration jobs are done. The BVS materials are resorbed over 6 months to 2 years, having a potential of eliminating chronic inflammation and enabling endothelial regeneration, when compared to permanent metallic stents. However, the clinical performances of existing BVS are inferior to DES or BMS. Improved understanding of the failure mechanisms of existing BVS is essential for the design of new generation BVS with more superior clinical performances.

## Challenges and Future Perspectives

With advancements in stent technologies and implantation techniques, the event-free survival rate for stent patients, in particular those with the new generation DES, has kept improving in the last decade. Based on the overall satisfactory results from the new generation DES, it can be extremely challenging for any new stent design or material to demonstrate better effectiveness while being safe and worth enormous investments into innovation and translation. An additional barrier that hampers the translation of the new design or material to clinical applications lies in the lack of sufficient understanding of mechanisms underlying the materials interactions. Further refinement of existing DES has an excellent benefit-risk ratio. Therefore, future developments of permanent metallic stents such as new functional coating or topography strategies may take the advantage of the current DES platform, based off it for design and performance analysis.

To address the long-term concern about permanent metallic stents—an ongoing risk of restenosis or thrombosis arising from the implant site which persists for at least 20 years (Yamaji et al., 2010; Yamaji et al., 2016; Stone et al., 2019) occurring after the first year at a rate of approximately 2–3% per year—BVS composed of biodegradable polymers or metals holds great potentials. Hypothetically, BVS provides a temporary scaffold for selective vascular regeneration—a complete re-endothelialization and significant outward remodeling. Such regeneration ideally may prevent the adverse events, including strut fractures, loss of vessel compliance, vasomotion, maladaptive vascular remodeling, and development of late neoatherosclerosis (Stone et al., 2019). Currently, this dream of BVS is not close to reality. A step further towards the dream is to innovate biodegradable materials engineering and innovate designs for both stent structure and surface, with the goal of balancing short-term and long-term requirements for BVS in terms of mechanics, therapy and regeneration. In parallel, mechanisms underlying BVS-related vascular pathobiology should be defined in a synergistic manner.

Finally, an amazing array of stent functionalization strategies are available in the literatures. However, translation efforts of them into clinical practices remain very limited if not futile. Future efforts in this area may fall into two categories: 1) Stent design according to specific needs of a patient’s cohort (or a specialized preclinical model) for competitive efficacy and superior outcome. For example, the regeneration of distal vasculature is only desired in ischemic conditions. Another example is for cell-capture stents: the EPC quantity, quality and capability can vary greatly among patients, and thus *in situ* stent cellularization results can vary accordingly. In this case, custom design strategies may be necessary for a specific patient’s cohort. 2) Multiple signaling strategies with defined spatiotemporal regimes of regenerative signals.
